# Body as expression of psychiatric distress: insights from restrictive eating disorders, non-suicidal self-injuries, and suicide attempts

**DOI:** 10.3389/fpsyg.2025.1552907

**Published:** 2025-03-14

**Authors:** Diletta Cristina Pratile, Marika Orlandi, Martina Maria Mensi

**Affiliations:** ^1^Department of Brain and Behavioral Sciences, University of Pavia, Pavia, Italy; ^2^Child Neurology and Psychiatry Unit, IRCCS Mondino Foundation, Pavia, Italy

**Keywords:** adolescence, body, functioning, multimethod assessment, non-suicidal self-injuries, prevention, restrictive eating disorder, suicide attempt

## Abstract

**Introduction:**

Adolescence is a developmental period marked by vulnerabilities where psychological distress often manifests through the body. Restrictive Eating Disorders (REDs), Non-Suicidal Self-Injury (NSSI), and Suicide Attempts (SAs) represent distinct yet overlapping expressions of this phenomenon.

**Methods:**

This cross-sectional study compared 60 adolescents (20 for each group) aged 12–18 across these groups using a comprehensive multimethod assessment, including the Kiddie Schedule for Affective Disorders and Schizophrenia (K-SADS-PL), the Structured Clinical Interview for DSM-5 Personality Disorders (SCID-5-PD), and the Rorschach Performance Assessment System (R-PAS).

**Results:**

The RED group exhibited the highest obsessive-compulsive symptoms and distorted interpersonal representations linked to perfectionism and body image concerns. R-PAS scores highlighted disorganized thinking and maladaptive self and other perceptions. The NSSI group displayed significant borderline traits, emotion regulation deficits, and impressionistic responses, with elevated R-PAS indices reflecting interpersonal defensiveness and vulnerability to emotional distress. The SA group showed severe depressive symptoms, dysregulation, and impaired thought organization, with the lowest functional scores (CGAS). Across all groups, adverse childhood experiences and distorted interpretations of stimuli emerged as common factors, supporting shared vulnerability.

**Discussion:**

This study provides a nuanced understanding of bodily expressions of psychological distress by integrating structured interviews, personality assessments, and performance-based tools. These findings emphasize the importance of tailored diagnostic and therapeutic strategies that address the unique and overlapping characteristics of these groups, advancing precision in adolescent mental health care.

## 1 Introduction

Adolescence is a pivotal developmental stage marked by significant physical, psychological, and social changes that shape identity and relationships while introducing vulnerabilities (Aliprandi et al., 2014; Ban and Ban, 2021). It represents a phase in which the body plays a central role in identity development and emotional regulation, a perspective emphasized in psychoanalytic research that explores the intricate relationship between mind and body (Lemma, 2014). Bodily experiences are seen as fundamental in shaping psychological development, influencing areas such as body image, psychosomatic symptoms, and the impact of trauma (Van der Kolk, 2015). Moreover, body image concerns intersect with the formation of dietary habits. This factor plays a crucial role in the development and maintenance of eating disorders, as sociocultural influences contribute to body dissatisfaction and disordered eating behaviors. Studies have shown that adolescents underestimating their body weight often adopt less healthy dietary behaviors. In contrast, those dissatisfied with their body image and driven by thinness are more likely to engage in weight-loss-related eating patterns (Bodega et al., 2023).

During adolescence, psychological issues frequently manifest through the body (Blakemore, 2012; Juli and Juli, 2020), and this could be particularly evident in conditions such as Restrictive Eating Disorders (REDs), Non-Suicidal Self-Injury (NSSI), and suicidal attempts (SAs) (Hua et al., 2024). These conditions reflect adolescents' struggles with their identity and emotional regulation, revealing the central role of the body in expressing psychological distress (Juli and Juli, 2020).

Those groups involve severe emotional dysregulation and share common risk factors, including trauma exposure, difficulties with emotion regulation, and body image concerns. Each group represents a distinct manifestation of distress: the RED group allows us to examine emotional regulation through restrictive eating behaviors. In contrast, the NSSI group highlights chronic emotional pain expressed through self-harming actions. The SA group offers insight into extreme emotional dysregulation and tendencies toward death. By studying these groups together, we aim to compare how emotional distress is embodied differently, providing a more comprehensive understanding of the continuum between eating disorders, self-harm, and suicidal behaviors.

Psychoanalytic research on eating disorders suggests that unmet emotional needs and unconscious conflicts may be expressed through the body rather than verbally, highlighting the symbolic role of eating behaviors in psychological functioning. Similarly, in individuals with eating disorders, the body may become the tool with which they desperately attempt to gain mastery and control over their feelings (Mirabella et al., 2023).

The prevalence of these issues has risen alarmingly lately, with societal and environmental factors amplifying distress, such as social media and peer influence. For instance, restrictive eating patterns often stem from complex psychological and social pressures exacerbated by idealized body standards perpetuated online (Hornberger et al., 2021; Frieiro et al., 2022; Mora et al., 2022; Silén and Keski-Rahkonen, 2022). Body dissatisfaction has been identified as a key factor in the development and maintenance of eating disorders, with sociocultural influences playing a central role in fostering body dissatisfaction, which in turn increases the risk of bulimic pathology (Stice and Shaw, 2002). Addressing body image disturbances is essential for enhancing prevention and treatment interventions.

Similarly, NSSI is frequently used as a maladaptive coping strategy to deal with intense emotional distress (Brunner et al., 2014; Cipriano et al., 2017; De Luca et al., 2023). Recent research suggests that body image may represent a necessary but not sufficient risk factor for NSSI in adolescents and that treatment for NSSI should consider targeting body-related pathology in addition to emotion regulation (Muehlenkamp and Brausch, 2012). Difficulties in emotion regulation, particularly in managing negative emotions, have been consistently associated with suicidal ideation and attempts across ages and populations, emphasizing the need to consider individual differences in emotion regulation to enhance understanding and inform clinical interventions (Colmenero-Navarrete et al., 2022).

In the most severe cases, these issues lead to SAs, one of the main causes of death among adolescents (Lo et al., 2020; World Health Organization, 2024). Moreover, the pervasive influence of social media further exacerbates the issue, as exposure to self-harm-related content can normalize or even encourage such behaviors in vulnerable individuals (Memon et al., 2018).

Understanding the mechanisms by which adolescents externalize their suffering is critical to prescribe the most timely, person-centered, and effective treatments even for symptom pictures with comorbidities, as shown by Davico et al. (2019), who found that adolescents with anorexia nervosa (AN) who engage in NSSI may exhibit distinct clinical and personality characteristics compared to those without NSSI.

The frequent occurrence of self-harm in adolescence highlights the need for a deeper understanding of the underlying mechanisms to develop personalized and effective treatment approaches tailored to adolescents.

The first aim of this study is to describe the typical psychological and behavioral characteristics of three adolescent patient groups who express psychological distress through their bodies: those with REDs, those engaging in NSSI, and those who have attempted suicide.

The second aim is to identify shared and distinct features across these three groups of patients using a multimethod assessment approach (Hopwood and Bornstein, 2014), including self-reports, clinician-reports, clinical interviews, and performance-based tests. Focusing on the shared and unique patterns of bodily symptom expression, this research seeks to refine diagnostic frameworks, enhance personalized interventions, and contribute to a deeper understanding of adolescent psychopathology.

We hypothesize that the RED group will show high levels of obsessive-compulsive tendencies, with impairments in self-representation and rigid thinking patterns. The NSSI group is expected to demonstrate difficulties in interpersonal relationships. We anticipate severe emotional dysregulation and reality testing impairments in the SA group. While each group may differ in the specific nature of their bodily symptom expression—NSSI through self-inflicted harm, REDs through restriction-related symptoms, and SAs reflecting both physical and emotional distress—all three groups are expected to share a common tendency to express psychological distress somatically.

## 2 Materials and methods

### 2.1 Design

This observational cross-sectional study was approved by the Ethics Committee of Policlinico San Matteo in Pavia (P-20200055757). The study adhered to the principles outlined in the REporting of studies Conducted using Observational Routinely-collected health Data (RECORD) guidelines (Supplementary material). Written informed consent was obtained from all participants and their caregivers, ensuring they understood that participation was voluntary and could be discontinued at any time without a reason. The authors confirm that all procedures followed the ethical standards of national and institutional review boards, the World Medical Organization (1964) principles, and subsequent revisions (World Medical Association, 2025). To safeguard privacy, all data were pseudonymized and can be accessed through the Zenodo repository upon request (Mensi, 2024).

### 2.2 Participants

We evaluated 319 adolescents referred to the Child Neurology and Psychiatry Unit of the IRCCS Mondino Foundation in Pavia, Italy, between March 2020 and October 2024. The participants were inpatient, outpatient, and day hospital regimens. The assessment was the same for all participants and could last 1 day (day hospital regimen), 2 days (outpatients), or 7 days (inpatients). Patients with less severe symptoms or who already had territorial care accessed via outpatient or day hospital regimens to receive a psychodiagnostic assessment. More severe patients accessed inpatient regimens to set up medications and psychotherapeutic treatment or activate territorial services in preparation for discharge. We included adolescents aged 12 to 18. The aim of the study required three groups of participants. The first group consisted of adolescents who met the diagnostic features for REDs outlined in the Diagnostic and Statistical Manual of Mental Disorders (DSM-5) (American Psychiatric Association, 2022). Included diagnoses encompassed restrictive subtypes of AN, atypical anorexia nervosa (A-AN), avoidant/restrictive food intake disorder (ARFID), and other specified feeding or eating disorders with predominantly restrictive features.

To be included in the second group, participants were required to have a documented history of NSSI but no prior SAs. We assessed the presence of NSSI using clinical interviews, confirmed through the question “*Has subject engaged in Non-Suicidal Self-Injurious Behavior?*” (dichotomic response “Yes/No”), reported in the Columbia-Suicide Severity Rating Scale (C-SSRS) (Posner et al., 2011). We also confirmed the absence of previous SAs using the C-SSRS (dichotomic response “Yes/No”).

The third group consisted of adolescents with a confirmed history of SAs, which included actual and interrupted attempts, according to clinical interviews and the C-SSRS.

Comorbid psychiatric symptoms were assessed using the Kiddie-Schedule for Affective Disorders and Schizophrenia, Present and Lifetime Version (K-SADS-PL-DSM-5) (Kaufman et al., 2016), which identifies both subthreshold and suprathreshold psychiatric symptoms. Participants were assigned to one of the three study groups (REDs, NSSI, or SAs) based on their primary type of self-directed physical harm presentation at the time of assessment, and they were included only in one group to avoid biases. However, given the high comorbidity rates in adolescent psychopathology, some participants may have exhibited symptoms of other psychiatric conditions, which were recorded but not used for group classification.

**Figure 1:**
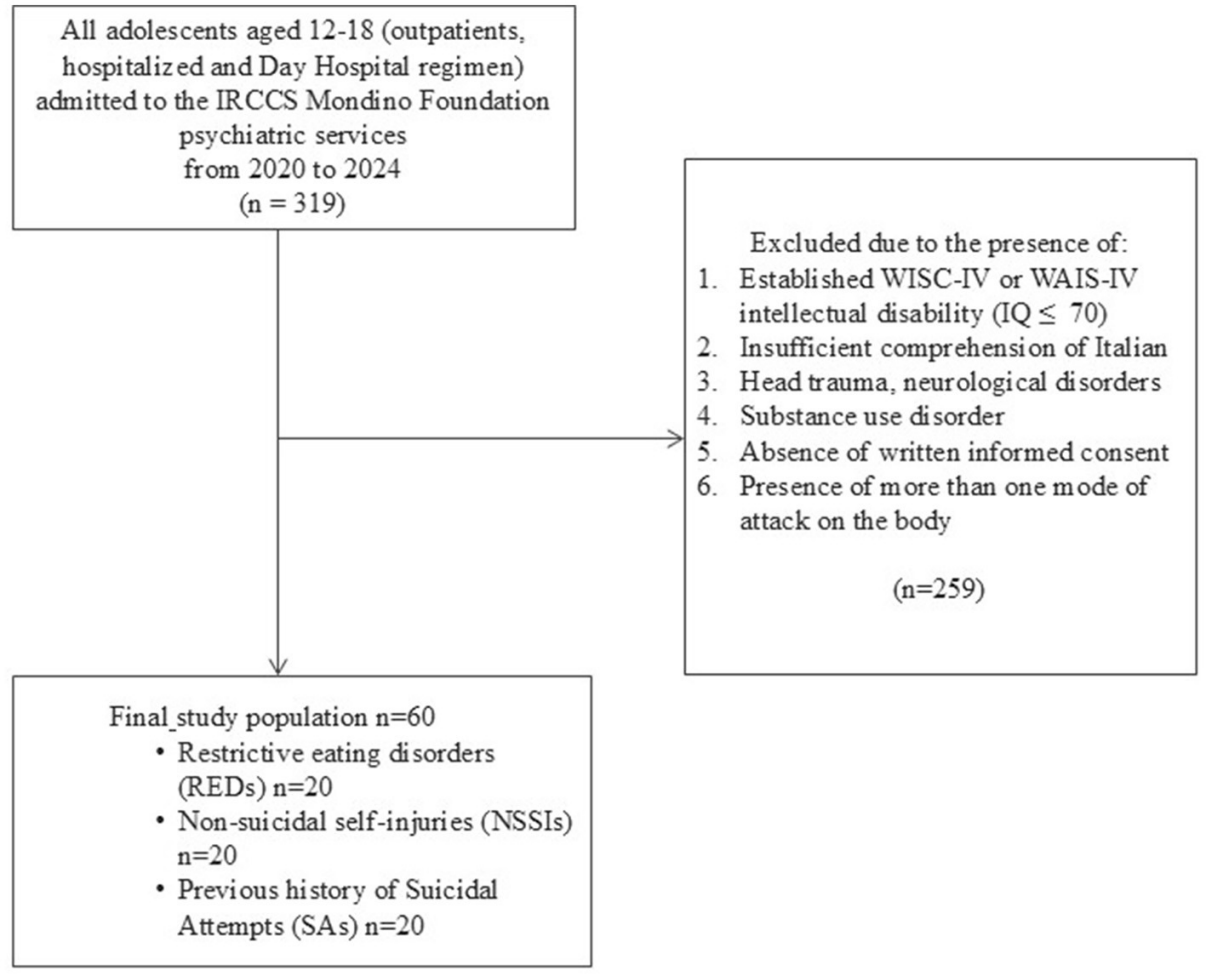
Study population flowchart.

We excluded individuals with: (I) intellectual disability (IQ ≤ 70) evaluated using the appropriate Wechsler intelligence scale (WISC-IV or WAIS-IV) (Wechsler, 2003, 2008); (II) inadequate proficiency in the Italian language; (III) a history of significant head trauma or the presence of any medical or neurological condition that could affect participation; and (IV) a diagnosis of substance use disorder. We also excluded those who refused to participate or provide written informed consent. Furthermore, individuals assigned to one group were excluded if they met inclusion criteria for any of the two other groups. The final study population included 60 participants divided into three groups of 20 individuals each. A detailed flowchart of the study population is provided in Figure 1.

### 2.3 Procedures

Clinicians conducted a comprehensive clinical evaluation utilizing a multimethod approach. This included:

- Sociodemographic data, including socioeconomic status (SES) (Hollingshead, 1975). SES levels were categorized as low (8–19), middle-low (20–29), middle (30–39), middle-high (40–54), and high (55–66).
- The Kiddie-Schedule for Affective Disorders and Schizophrenia, Present and Lifetime Version (K-SADS-PL-DSM-5) (Kaufman et al., 2016), a semi-structured interview to support the diagnoses and identify comorbidities. This tool has been translated and adapted into more than 20 languages, including Italian (Kaufman et al., 2019), with fair-to-excellent reliability, validity, test-retest reliability, and a high interrater agreement (Kaufman et al., 1997).
- The Structured Clinical Interview for DSM-5 Personality Disorders (SCID-5-PD) (First et al., 2017), a semi-structured interview administered to participants aged 14 and older, following the initial self-report questionnaire filled in by individuals. This tool categorically assesses the presence (subthreshold or suprathreshold) or absence of personality disorder traits based on DSM-5 criteria. The Italian version is standardized and has strong inter-rater reliability (Somma et al., 2020).
- The Columbia-Suicide Severity Rating Scale (C-SSRS) (Posner et al., 2011). A semi-structured interview that assesses suicidal ideation, previous history of actual (“*Have you made a suicide attempt?*”), interrupted (“*Has there been a time when you started to do something to end your life but someone or something stopped you before you actually did anything?”*), and aborted (“*Has there been a time when you started to do something to try to end your life but you stopped yourself before you actually did anything?*”) SAs (dichotomic response “Yes/No”), and the presence of NSSI (“*Has subject engaged in Non-Suicidal Self-Injurious Behavior?*”) (dichotomic response “Yes/No”). It is available and validated in more than 150 languages, and studies demonstrated evidence of moderate to strong reliability (Nam et al., 2024).
- The Clinical Global Impression-Severity scale (CGI-S) (Guy, 1976), a valid and reliable (Berk et al., 2008) clinician-report measure designed to capture symptoms' overall intensity and severity on an 8-point scale from 0 (no assessment) to 7 (between the most impaired patients).
- The Children's Global Assessment Scale (CGAS) (Shaffer et al., 1983), a clinician-report scale that provides standardized ratings of patients' overall functioning in social and occupational domains on a 100-point rating scale from 0 (highly impaired) to 100 (excellent functioning). It is reliable between raters and across time (Shaffer et al., 1983), with moderate inter-rater reliability (Lundh et al., 2010).
- The maximum-performance Wechsler Intelligence Scale for Children (WISC-IV) (Wechsler, 2003) or the Wechsler Adult Intelligence Scale (WAIS-IV) (Wechsler, 2008) were administered, depending on the participant's age, to evaluate cognitive functioning and exclude participants with intellectual disability. The test highlights total IQ, verbal comprehension index (VCI), perceptual reasoning index (PRI), working memory index (WMI), and processing speed index (PSI). Those performance-based tests have demonstrated strong psychometric properties, such as validity and reliability, in different languages, including Italian (Orsini et al., 2012; Pezzuti et al., 2018; Kush and Canivez, 2019; Andrikopoulos, 2021).
- The Rorschach inkblot test was administered following the Rorschach Performance Assessment System (R-PAS) method (Meyer et al., 2011). This typical performance-based tool engages participants in a perceptual and interpretive task using ten standardized inkblot cards. The responses are systematically analyzed to reveal insights into the individual's psychological functioning and personality traits. The R-PAS methodology is recognized for its modernized approach, strong psychometric properties, and robust validity and reliability, supported by international research and evidence (Mihura et al., 2013; Giromini et al., 2015; Viglione et al., 2015, 2022; Pignolo et al., 2017).

### 2.4 Plan of analysis

Statistical analyses were performed using JASP software, version 0.19.3.0. Descriptive statistics were performed for demographic and clinical data of the overall sample and each subgroup separately. Comparative analyses were conducted to examine differences across the three groups. The threshold for statistical significance was set at α = 0.05. Statistical analyses were performed using both non-parametric and parametric approaches to ensure robustness in the findings, especially due to the small sample sizes and the potential violation of normality assumptions. Initially, to avoid potential bias from small and non-homogeneous sample sizes, the Kruskal-Wallis test was conducted to compare the differences between the groups. The Kruskal-Wallis test is a non-parametric alternative to ANOVA, which does not assume normality and is suitable for non-normally distributed data. *Post-hoc* analyses for the significative comparisons between the three groups using the Dunn test. To minimize the risk of type I errors associated with multiple comparisons, a Bonferroni correction was applied to all *post-hoc* analyses.

However, we performed an ANCOVA to explore the potential influence of gender as a covariate on the group differences. ANCOVA allows for a more refined analysis by controlling for variables that may affect the dependent variables, thus providing a clearer understanding of the effect of the independent variables. In both the Kruskal-Wallis test and the ANCOVA, a significance level of *p* < 0.05 was used to determine statistical significance. Finally, we also report the fixed omega squared (ω^2^), a robust effect size measure suitable for small samples (Kroes and Finley, 2023). Thresholds indicate very small (< 0.01), small (between ≤ 0.01 and < 0.06), medium (between ≤ 0.06 and < 0.14), and large effect (≥0.14) and quantify the magnitude of the observed group differences.

## 3 Results

The study included 60 participants (55 females; mean age = 15.55 years, SD = 1.71), divided into three groups: 20 patients with REDs (19 females; mean age = 15.49 years, SD = 2.01), 20 presenting NSSI (18 females; mean age = 15.67 years, SD = 1.69), and 20 with a previous history of SA (18 females; mean age = 15.49 years, SD = 1.48). They did not differ in age (*p* = 0.846), gender (*p* = 0.804), SES score (*p* = 0.633), ethnicity (*p* = 0.419), birth order (*p* = 0.333), number of siblings (*p* = 0.220), adoption (*p* = 0.765), or separated/divorced parents (*p* = 0.660). Sociodemographic data is presented in Table 1.

**Table 1:** Sociodemographic data.

**Variable**	**Total *N* (%)**	**REDs *N* (%)**	**NSSIs *N* (%)**	**SAs *N* (%)**	***p***
Age (mean ± SD)	15.55 ± 1.71	15.49 ± 2.01	15.67 ± 1.69	15.49 ± 1.48	0.846
Gender (female %)	55 (91.67)	19 (95)	18 (90)	18 (90)	0.804
SES^a^ (mean ± SD)	34.52 ± 13.54	35.03 ± 14.56	34.13 ± 11.04	34.36 ± 15.11	0.633
**Ethnicity**					0.419
Caucasian	52 (86.66)	18 (90)	16 (80)	18 (90)	
Asian	1 (1.67)	0 (0)	0 (0)	1 (5)	
African	3 (5)	0 (0)	2 (10)	1 (5)	
Latina	3 (5)	1 (5)	2 (10)	0 (0)	
Mixed	1 (1.67)	1 (5)	0 (0)	0 (0)	
**Siblings**					0.333
Only child	12 (20)	6 (30)	2 (10)	4 (20)	
Elder siblings	26 (43.33)	10 (50)	7 (35)	9 (45)	
Younger siblings	17 (28.33)	4 (20)	8 (40)	5 (25)	
Elder and younger siblings	5 (8.33)	0 (0)	3 (15)	2 (10)	
**Number of siblings**					0.220
0	12 (20)	6 (30)	2 (10)	4 (20)	
1	34 (56.66)	13 (65)	11 (55)	10 (50)	
2	13 (21.67)	1 (5)	6 (30)	6 (30)	
3	1 (1.67)	0 (0)	1 (5)	0 (0)	
Adopted	4 (6.67)	1 (5)	2 (10)	1 (5)	0.765
Separated/divorced parents	20 (33.33)	7 (35)	8 (40)	5 (25)	0.660

Significance: ^*^p < 0.05; ^a^SES: low (8–19), middle-low (20–29), middle (30–39), middle-high (40–54), and high (55–66).

Table 2 reports frequencies of K-SADS-PL and SCID-5-PD subthreshold and suprathreshold scores in the three subgroups. All groups showed similar PTSD scores on the K-SADS-PL. Data showed that in the RED group, 85% of participants exhibited depression symptoms on the K-SADS-PL, and many also showed subthreshold separation anxiety. Moreover, 95% of this group displayed symptoms indicative of REDs. Also, nearly half of the RED group exhibited subthreshold OCD symptoms.

**Table 2:** Frequencies of K-SADS-PL and SCID-5-PD subthreshold and suprathreshold scores in the three subgroups.

**Variables**		**REDs** ***N*** **(%)**			**NSSIs** ***N*** **(%)**			**SAs** ***N*** **(%)**		
		**No**	**Sub**	**Supra**	**No**	**Sub**	**Supra**	**No**	**Sub**	**Supra**
**K-SADS-PL**	Depression	3 (15)	17 (85)	0 (0)	0 (0)	20 (100)	0 (0)	0 (0)	5 (25)	15 (75)
	Mania	19 (95)	1 (5)	0 (0)	16 (80)	4 (20)	0 (0)	16 (80)	3 (15)	1 (5)
	Hypomania	18 (90)	2 (10)	0 (0)	17 (85)	3 (15)	0 (0)	19 (95)	1 (5)	0 (0)
	Dysregulation	19 (95)	1 (5)	0 (0)	18 (90)	2 (10)	0 (0)	13 (65)	5 (25)	2 (10)
	Psychosis	14 (70)	6 (30)	0 (0)	12 (60)	8 (40)	0 (0)	7 (35)	11 (55)	2 (10)
	Panic	9 (45)	11 (55)	0 (0)	10 (50)	10 (50)	0 (0)	12 (60)	4 (20)	4 (20)
	Agoraphobia	16 (80)	4 (20)	0 (0)	18 (90)	2 (10)	0 (0)	14 (70)	5 (25)	1 (5)
	Separation anxiety	12 (60)	8 (40)	0 (0)	18 (90)	2 (10)	0 (0)	13 (65)	6 (30)	1 (5)
	Social anxiety	13 (65)	7 (35)	0 (0)	8 (40)	12 (60)	0 (0)	7 (35)	6 (30)	7 (35)
	Phobia	12 (60)	8 (40)	0 (0)	17 (85)	3 (15)	0 (0)	15 (75)	5 (25)	0 (0)
	GAD	11 (55)	9 (45)	0 (0)	12 (60)	8 (40)	0 (0)	7 (35)	5 (25)	8 (40)
	OCD	11 (55)	9 (45)	0 (0)	18 (90)	2 (10)	0 (0)	16 (80)	3 (15)	1 (5)
	AN	1 (5)	18 (90)	1 (5)	14 (70)	6 (30)	0 (0)	14 (70)	4 (20)	2 (10)
	ADHD	19 (95)	1 (5)	0 (0)	19 (95)	1 (5)	0 (0)	18 (90)	2 (10)	0 (0)
	ODD	19 (95)	1 (5)	0 (0)	17 (85)	3 (15)	0 (0)	16 (80)	3 (15)	1 (5)
	Conduct disorder	19 (95)	1 (5)	0 (0)	17 (85)	3 (15)	0 (0)	20 (100)	0 (0)	0 (0)
	PTSD	17 (85)	3 (15)	0 (0)	17 (85)	3 (15)	0 (0)	15 (75)	2 (10)	3 (15)
**SCID-5-PD** ^**a**^	Avoidant	13 (65)	2 (10)	2 (10)	13 (65)	5 (25)	1 (5)	13 (65)	1 (5)	4 (20)
	Dependent	14 (70)	2 (10)	1 (5)	19 (95)	0 (0)	0 (0)	18 (90)	0 (0)	0 (0)
	Obsessive-Compulsive	10 (50)	4 (20)	3 (15)	15 (75)	0 (0)	4 (20)	10 (50)	0 (0)	8 (40)
	Paranoid	17 (85)	0 (0)	0 (0)	18 (90)	1 (5)	0 (0)	16 (80)	1 (5)	1 (5)
	Schizotypal	15 (75)	2 (10)	0 (0)	17 (85)	2 (10)	0 (0)	17 (85)	0 (0)	1 (5)
	Schizoid	17 (85)	0 (0)	0 (0)	18 (90)	1 (5)	0 (0)	18 (90)	0 (0)	0 (0)
	Histrionic	17 (85)	0 (0)	0 (0)	18 (90)	1 (5)	0 (0)	18 (90)	0 (0)	0 (0)
	Narcissistic	17 (85)	0 (0)	0 (0)	19 (95)	0 (0)	0 (0)	17 (85)	1 (5)	0 (0)
	Borderline	14 (70)	2 (10)	1 (5)	5 (25)	6 (30)	8 (40)	10 (50)	1 (5)	7 (35)
	Antisocial	17 (85)	0 (0)	0 (0)	17 (85)	1 (5)	1 (5)	17 (85)	0 (0)	1 (5)

^a^Only administered to patients from 14 y.o. on. ADHD, Attention Deficit Hyperactivity Disorder; AN, anorexia nervosa; GAD, generalized anxiety disorder; OCD, obsessive-compulsive disorder; ODD, oppositional defiant disorder; PTSD, post-traumatic stress disorder.

In the NSSI group, all participants had subthreshold depressive symptoms. Moreover, 50% of NSSIs exhibited subthreshold panic symptoms, and 60% showed subthreshold social anxiety symptoms. The SCID-5-PD revealed that 70% of NSSIs exhibited borderline personality traits.

The SA group had the highest frequency of suprathreshold scores across K-SADS-PL domains, with all participants showing depressive symptoms. Notably, 75% of these participants scored in the suprathreshold range for depression. This group also demonstrated the highest prevalence of emotional dysregulation (35%), along with elevated rates of psychotic symptoms, social anxiety, and generalized anxiety disorder (GAD).

Table 3 presents the means, standard deviations, group comparisons, and *post-hoc* analyses for scores on the K-SADS-PL, SCID-5-PD, Wechsler scales, CGI-S, and CGAS. Table 4 shows ANCOVA results. Data showed that SAs had higher depressive symptom scores than other groups in both non-parametric and parametric analyses (*p* < 0.001), with a large effect size. Furthermore, the SA group had significantly higher dysregulation symptoms (non-parametric: *p* = 0.030; parametric: *p* = 0.017) and social anxiety scores (non-parametric: *p* = 0.021; parametric: *p* = 0.005) than the RED group. Those results are supported by large and medium effect sizes, respectively. The RED group had statistically significantly higher AN scores than the other groups (non-parametric: *p* < 0.001; parametric: *p* < 0.001), supported by a large effect size. The SA group also had higher GAD scores than NSSIs (non-parametric: *p* = 0.038; parametric: *p* = 0.007). ANCOVA found that the SA group had higher GAD scores than REDs (*p* = 0.011), with a large effect size. Moreover, while non-parametric analysis indicated significantly higher OCD scores in the RED group than in NSSIs (*p* = 0.039; small effect size), ANCOVA did not confirm this difference. Conversely, ANCOVA found that the SA group had significantly higher psychosis symptom scores than the RED group (*p* = 0.042).

**Table 3:** Comparisons of psychodiagnostic instruments in the three groups and *post-hoc* analyses corrected for the Bonferroni test.

**Variables**		**REDs** ***N** **=*** **20**		**NSSIs** ***N** **=*** **20**		**SAs** ***N** **=*** **20**		**F**	***p***	**REDs vs. NSSIs**	**REDs vs. SAs**	**NSSIs vs. SAs**	**Contrast**
		***M***	***SD***	***M***	***SD***	***M***	***SD***			***p***	***p***	***p***	
**K-SADS-PL**	Depression	0.850	0.366	1.000	0.000	1.750	0.444	36.832	< 0.001^***^	1.000	< 0.001^***^	< 0.001^***^	a < c; b < c
	Mania	0.050	0.224	0.200	0.410	0.250	0.550	2.351	0.309	-	-	-	-
	Hypomania	0.100	0.308	0.150	0.366	0.050	0.224	1.093	0.579	-	-	-	-
	Dysregulation	0.050	0.224	0.100	0.308	0.450	0.686	7.658	0.022^*^	1.000	0.030^*^	0.090	a < c
	Psychosis	0.300	0.470	0.400	0.503	0.750	0.639	6.161	0.046^*^	1.000	0.051	0.223	-
	Panic	0.550	0.510	0.500	0.513	0.600	0.821	0.101	0.951	-	-	-	-
	Agoraphobia	0.200	0.410	0.100	0.308	0.350	0.587	2.617	0.270	-	-	-	-
	Separation anxiety	0.400	0.503	0.100	0.308	0.400	0.503	5.001	0.082	-	-	-	-
	Social anxiety	0.350	0.587	0.600	0.821	1.000	0.858	7.301	0.026^*^	0.560	0.021^*^	0.502	a < c
	Phobia	0.400	0.503	0.150	0.366	0.250	0.444	3.185	0.203	-	-	-	-
	GAD	0.450	0.510	0.400	0.503	1.050	0.887	7.521	0.023^*^	1.000	0.076	0.038^*^	b < c
	OCD	0.450	0.510	0.100	0.308	0.250	0.550	6.441	0.040^*^	0.039^*^	0.276	1.000	a > b
	AN	1.000	0.324	0.300	0.470	0.400	0.681	19.198	< 0.001^***^	< 0.001^***^	< 0.001^***^	1.000	a > b; a>c
	ADHD	0.050	0.224	0.050	0.224	0.100	0.308	0.527	0.768	-	-	-	-
	ODD	0.050	0.224	0.150	0.366	0.250	0.550	2.069	0.355	-	-	-	-
	Conduct disorder	0.050	0.224	0.150	0.366	0.000	0.000	3.687	0.158	-	-	-	-
	PTSD	0.158	0.375	0.150	0.366	0.400	0.754	1.211	0.546	-	-	-	-
**IQ**	Tot	110.474	14.230	103.25	18.756	102.55	17.998	2.817	0.244	-	-	-	-
	VCI	111.263	16.107	106.85	16.693	108.474	19.486	1.001	0.606	-	-	-	-
	PRI	112.842	14.416	107.90	15.586	107.737	16.193	1.881	0.390	-	-	-	-
	WMI	95.158	12.13	92.20	20.493	87.105	12.00	3.425	0.180	-	-	-	-
	PSI	106.895	18.138	97.75	20.414	96.222	19.839	2.353	0.308	-	-	-	-
**SCID-5-PD** ^**a**^	Avoidant	0.353	0.702	0.368	0.597	0.500	0.857	0.201	0.905	-	-	-	-
	Dependent	0.235	0.562	0.000	0.000	0.000	0.000	6.781	0.034^*^	0.067	0.072	1.000	-
	Obsessive-Compulsive	0.588	0.795	0.421	0.838	0.889	1.023	2.440	0.295	-	-	-	-
	Paranoid	0.000	0.000	0.053	0.229	0.167	0.514	2.063	0.356	-	-	-	-
	Schizotypal	0.188	0.332	0.105	0.315	0.111	0.471	0.357	0.837	-	-	-	-
	Schizoid	0.000	0.000	0.053	0.229	0.000	0.000	1.842	0.398	-	-	-	-
	Histrionic	0.000	0.000	0.053	0.229	0.000	0.000	1.842	0.398	-	-	-	-
	Narcissistic	0.000	0.000	0.000	0.000	0.056	0.236	2.000	0.368	-	-	-	-
	Borderline	0.235	0.562	1.158	0.834	0.833	0.985	10.333	0.006^**^	0.004^**^	0.165	0.606	a < b
	Antisocial	0.000	0.000	0.158	0.501	0.111	0.471	1.825	0.402	-	-	-	-
	**CGI-S**	4.200	1.281	4.600	0.754	4.700	0.979	3.322	0.190	-	-	-	-
	**CGAS**	56.600	11.491	49.700	8.640	45.600	12.172	8.527	0.014^*^	0.212	0.012^*^	0.836	a > c

Significance: ^*^p < 0.05; ^**^p < 0.01; ^**^p < 0.001. ^a^Only administered to patients from 14 y.o. on. Groups: a, REDs; b, NSSIs; c, SAs. ADHD, Attention Deficit Hyperactivity Disorder; AN, anorexia nervosa; CGAS, Children's Global Assessment Scale; CGI-S, Clinical Global Impression-Severity; GAD, generalized anxiety disorder; OCD, obsessive-compulsive disorder; ODD, oppositional defiant disorder; PRI, perceptual reasoning index; PSI, processing speed index; PTSD, post-traumatic stress disorder; VCI, verbal comprehension index; WMI, working memory index.

**Table 4:** ANCOVA and effect size concerning psychodiagnostic instruments in the three groups.

**Variables**		***F***	***p***	***ω^2^***	**REDs vs. NSSIs**	**REDs vs. SAs**	**NSSIs vs. SAs**	**Contrast**
					***p***	***p***	***p***	
**K-SADS-PL**	Depression	41.631	< 0.001^***^	0.578	0.458	< 0.001^***^	< 0.001^***^	a < c; b < c
	Mania	6.465	0.341	0.003	-	-	-	-
	Hypomania	0.536	0.588	0.000	-	-	-	-
	Dysregulation	4.841	0.012^*^	0.113	1.000	0.017^*^	0.052	a < c
	Psychosis	3.633	0.033^*^	0.080	1.000	0.042^*^	0.135	a < c
	Panic	0.133	0.876	0.000	-	-	-	-
	Agoraphobia	1.537	0.224	0.018	-	-	-	-
	Separation anxiety	2.574	0.085	0.050	-	-	-	-
	Social anxiety	5.505	0.007^**^	0.130	0.577	0.005^**^	0.157	a < c
	Phobia	1.459	0.241	0.015	-	-	-	-
	GAD	6.465	0.003^**^	0.150	1.000	0.011^*^	0.007^**^	a < c; b < c
	OCD	2.605	0.083	0.051	-	-	-	-
	AN	10.437	< 0.001^**^	0.237	< 0.001^***^	0.002^**^	1.000	a > b; a > c
	ADHD	0.227	0.798	0.000	-	-	-	-
	ODD	1.185	0.313	0.006	-	-	-	-
	Conduct disorder	1.903	0.159	0.029	-	-	-	-
	PTSD	1.491	0.234	0.016	-	-	-	-
**IQ**	Tot	1.240	0.297	0.008	-	-	-	-
	VCI	0.349	0.707	0.000	-	-	-	-
	PRI	0.599	0.533	0.000	-	-	-	-
	WMI	1.529	0.226	0.017	-	-	-	-
	PSI	1.508	0.231	0.018	-	-	-	-
**SCID-5-PD** ^**a**^	Avoidant	0.245	0.784	0.000	-	-	-	-
	Dependent	3.106	0.054	0.073	-	-	-	-
	Obsessive-Compulsive	1.45	0.253	0.015	-	-	-	-
	Paranoid	1.243	0.297	0.009	-	-	-	-
	Schizotypal	0.009	0.991	0.000	-	-	-	-
	Schizoid	0.918	0.406	0.000	-	-	-	-
	Histrionic	0.009	0.991	0.000	-	-	-	-
	Narcissistic	1.006	0.373	0.000	-	-	-	-
	Borderline	6.573	0.003^**^	0.165	0.002^**^	0.068	0.683	a < b
	Antisocial	0.595	0.555	0.000	-	-	-	-
	**CGI-S**	1.281	0.286	0.009	-	-	-	-
	**CGAS**	4.944	0.011^*^	0.116	0.177	0.009^**^	0.716	a > c

Significance: ^*^p < 0.05; ^**^p < 0.01; ^***^p < 0.001; ω^2^: very small (< 0.01), small (between ≤ 0.01 and < 0.06), medium (between ≤ 0.06 and < 0.14), large effect (≥0.14). ^a^Only administered to patients from 14 y.o. on. Groups: a, REDs; b, NSSIs; c, SAs. ADHD, Attention Deficit Hyperactivity Disorder; AN, anorexia nervosa; CGAS, Children's Global Assessment Scale; CGI-S, Clinical Global Impression-Severity; GAD, generalized anxiety disorder; OCD, obsessive-compulsive disorder; ODD, oppositional defiant disorder; PRI, perceptual reasoning index; PSI, processing speed index; PTSD, post-traumatic stress disorder; VCI, verbal comprehension index; WMI, working memory index.

Concerning the SCID-5-PD, the NSSI group expressed statistically higher borderline personality traits than the RED group (Kruskal-Wallis: *p* = 0.004; ANCOVA: *p* = 0.002), with a large effect size. The RED group expressed significantly better functioning assessed through CGAS than the SA group (non-parametric: *p* = 0.012; parametric: *p* = 0.009), with a large effect size. No differences were found in IQ indexes and CGI-S scores.

Concerning R-PAS, all groups scored two standard deviations above average on Form Quality minus (FQ-) and one standard deviation above average on Whole or Detailed response with form quality minus (WD-). Those indexes refer to distortions or misinterpretations of stimuli present in atypical contexts and simple, familiar, and easily interpreted contexts.

The RED group had elevated Ego Impairment Index-3 (EII-3) and Thought & Perception Composite (TP-Comp) scores alongside low Form Quality Ordinary (FQo). Those indicate difficulties with reality examination, the possible presence of thought disorder, disturbing content, and relational distress. Additionally, they scored high on the Proportion of Poor or Good Human Representation (PHR/GPHR), an index showing that the representation of self and/or others is problematic due to distortions, confusion, malevolence, aggression, personalization, partial, unrealistic, or vulnerable views. Similarly, results highlighted high Unusual Detail (Dd) scores, pointing to a tendency to examine rare, minor, or idiosyncratic details, wanting to impose one's point of view, and Anatomy (An) scores, highlighting the presence of physical, medical, or body-related concerns, along with fragility or vulnerability of body image or mind.

The NSSI group scored two standard deviations above average on the EII-3 and TP-Comp and scored low on the FQo index. Moreover, they had high scores on Human movements with form quality minus (M-), highlighting atypical and distorted understanding of others that suggests the presence of disturbed interpersonal relationships, Personal responses (PER), indicating relationship defensiveness, and an indexes.

The SA group scored above average on both the EII-3 and TP-Comp indexes.

Table 5 shows the means, standard deviations, comparisons between all R-PAS indexes in the three groups, and *post-hoc* analyses. Table 6 shows ANCOVA results. The RED group scored higher on Pull (Pu) than the NSSI group (non-parametric: *p* = 0.037; parametric: *p* = 0.025), showing a medium effect size. The Pu index is linked to the efforts to impress or please the examiner, reflecting ambition or productivity as a defense against anxiety or insecurity. The NSSI group showed significantly higher PER scores than the RED group in Kruskal-Wallis (*p* = 0.034) and ANCOVA (*p* = 0.020) analyses, with a medium effect size. The RED group had significantly higher scores in the NPH/SumH index than the NSSI group (non-parametric: *p* = 0.039; parametric: *p* = 0.017), with a medium effect size. The NPH/SumH indicates unrealistic or overly fanciful representations of interpersonal relationships. The Kruskal-Wallis test highlighted that the NSSI group had significantly higher scores in Vague (Vg%) than the SA group (*p* = 0.043), with a medium effect size. The Vg% index suggests impressionistic, evasive, defensive responses and engagement reluctance. The NSSI group also scored statistically higher on Color Blend (CBlend) (*p* = 0.011) and Critical Contents (CritCont%) (*p* = 0.024) than the RED group. The CBlend index reflects an individual's attraction to uncertainty and ambiguity, highlighting a tendency to avoid spontaneous, positive emotions, which may evoke discomfort. The CritCont% index may indicate perceived traumatic experiences, dissociative tendencies, a weakened ego censoring mechanism, or exaggerating psychopathology to shock the examiner. The captions of all R-PAS variables are shown in Supplementary Table S1.

**Table 5:** Comparisons between the R-PAS variables in the three groups and Bonferroni *post-hoc* analyses.

	**REDs** ***N** **=*** **20**		**NSSIs** ***N** **=*** **20**		**SAs** **N** **=** **20**		**F**	***p***	**REDs vs. NSSIs**	**REDs vs. SAs**	**NSSIs vs. SAs**	**Contrast**
	***M***	***SD***	***M***	***SD***	***M***	***SD***			***p***	***p***	***p***	
**Page 1**												
**Administration. behaviors and observation**												
Pr	97.750	10.497	102.850	13.453	99.800	12.887	1.358	0.507	-	-	-	-
Pu	109.050	14.877	98.450	7.681	108.300	14.068	7.865	0.020^*^	0.037^*^	1.000	0.053	a > b
CT	107.750	14.935	105.450	15.743	105.350	15.260	0.150	0.928	-	-	-	-
**Engagement and cognitive processing**												
Complexity	102.850	11.829	100.950	15.813	94.700	18.494	1.997	0.368	-	-	-	-
R	110.600	13.717	103.800	14.681	104.350	11.513	1.763	0.414	-	-	-	-
F%	99.900	14.201	99.550	13.328	104.000	10.892	1.756	0.416	-	-	-	-
Blend	98.300	11.585	105.800	8.377	99.250	13.158	3.561	0.169	-	-	-	-
Sy	96.400	13.430	103.150	10.499	97.150	12.304	3.950	0.139	-	-	-	-
MC	99.100	11.986	104.850	13.709	103.350	25.668	3.258	0.196	-	-	-	-
MC-PPD	96.600	17.440	103.350	11.037	99.400	13.949	1.525	0.467	-	-	-	-
M	99.900	12.553	105.200	12.340	101.900	15.691	2.883	0.237	-	-	-	-
M/MC	100.529	14.770	104.813	7.250	104.357	12.351	1.983	0.371	-	-	-	-
(CF+C)/SumC	102.800	12.182	106.750	16.096	96.889	18.292	1.199	0.549	-	-	-	-
**Perception and thinking problems**												
EII-3	118.850	18.380	123.350	22.876	118.950	17.431	0.289	0.866	-	-	-	-
TP-Comp	117.150	18.048	120.800	20.411	117.400	14.723	0.169	0.919	-	-	-	-
WSumCog	102.250	14.952	109.400	15.299	103.300	10.781	3.577	0.167	-	-	-	-
SevCog	103.300	14.305	108.750	13.695	104.300	11.948	1.905	0.386	-	-	-	-
FQ-%	121.650	18.936	120.800	18.808	123.200	16.957	0.089	0.956	-	-	-	-
WD-%	113.800	15.117	115.950	20.710	113.500	19.321	0.051	0.975	-	-	-	-
FQo%	83.900	13.118	82.950	15.405	92.000	12.740	4.441	0.109	-	-	-	-
P	91.300	14.368	96.650	13.264	92.150	12.893	0.555	0.758	-	-	-	-
**Stress and distress**												
YTVC'	104.550	18.696	101.400	13.975	105.800	10.948	0.558	0.756	-	-	-	-
m	103.684	11.629	101.550	8.519	95.900	25.020	2.281	0.320	-	-	-	-
Y	107.750	14.531	109.400	11.971	107.000	12.637	0.612	0.736	-	-	-	-
MOR	101.000	9.712	109.700	13.777	106.750	12.139	4.169	0.124	-	-	-	-
SC-Comp	98.231	12.663	101.857	14.501	103.077	12.919	0.796	0.672	-	-	-	-
**Self and other representation**												
ODL%	91.550	14.468	98.550	12.484	100.450	13.461	4.533	0.104	-	-	-	-
SR	100.700	13.055	103.850	14.908	107.450	13.555	2.441	0.295	-	-	-	-
MAP/MAHP	NA	NA	NA	NA	NA	NA	NA	NA	-	-	-	-
PHR/GPHR	111.368	16.153	109.941	16.660	107.778	14.799	0.368	0.832	-	-	-	-
M-	109.150	12.762	112.550	16.732	106.900	13.954	1.424	0.491	-	-	-	-
AGC	95.450	13.109	99.500	12.403	100.700	17.159	0.885	0.642	-	-	-	-
H	99.000	13.853	107.000	7.636	101.100	11.073	4.185	0.123	-	-	-	-
COP	100.650	9.422	108.900	11.836	106.100	12.985	5.471	0.065	-	-	-	-
MAH	98.350	7.191	102.450	10.802	103.550	10.986	2.156	0.340	-	-	-	-
**Page 2**												
**Engagement and cognitive processing**												
W%	91.850	10.530	97.800	14.799	95.000	8.367	1.228	0.541	-	-	-	-
Dd%	112.100	10.677	103.600	12.584	109.100	12.303	3.588	0.166	-	-	-	-
SI	103.350	14.662	97.100	14.821	102.600	13.682	1.977	0.372	-	-	-	-
IntCont	100.250	11.666	102.750	12.665	105.500	11.727	1.362	0.506	-	-	-	-
Vg %	100.600	7.133	103.100	8.546	97.500	7.633	6.001	0.050^*^	0.771	0.551	0.043^*^	b>c
V	99.550	7.830	101.550	8.338	104.200	14.118	2.868	0.238	-	-	-	-
FD	102.300	9.750	105.650	10.830	108.000	9.061	3.872	0.144	-	-	-	-
R8910%	99.250	9.792	98.800	9.384	97.950	11.936	0.330	0.848	-	-	-	-
WSumC	102.400	12.873	103.550	12.717	98.550	8.769	3.644	0.162	-	-	-	-
C	102.550	10.318	104.900	11.271	100.300	8.578	1.591	0.451	-	-	-	-
Mp/(Ma+Mp)	99.273	17.533	92.308	16.388	99.222	16.146	0.967	0.616	-	-	-	-
**Perception and thinking problems**												
FQu%	101.350	16.512	101.050	18.917	92.150	10.449	3.624	0.163	-	-	-	-
**Stress and distress**												
PPD	100.600	17.751	103.050	7.756	100.400	12.462	0.512	0.774	-	-	-	-
CBlend	98.400	7.052	105.350	1.179	96.000	21.978	9.513	0.009^**^	0.011^**^	1.000	0.052	a < b
C'	106.250	18.238	95.600	9.955	102.750	10.740	5.217	0.074	-	-	-	-
CritCont%	100.450	11.749	112.550	16.401	105.450	13.340	7.047	0.029^*^	0.024^*^	0.510	0.582	a < b
**Self and other representation**												
SumH	102.850	12.279	101.100	10.882	99.400	10.262	0.660	0.719	-	-	-	-
NPH/SumH	106.000	17.923	91.647	11.651	101.000	14.604	6.520	0.038^*^	0.039^*^	1.000	0.210	a > b
V-Comp	101.100	12.973	98.500	10.875	98.050	11.441	0.883	0.643	-	-	-	-
r	105.150	12.795	106.950	14.373	105.650	13.511	0.137	0.934	-	-	-	-
p/(a+p)	98.000	18.107	97.000	14.661	101.125	17.843	0.784	0.676	-	-	-	-
AGM	99.050	9.058	107.050	15.219	103.450	12.185	3.133	0.209	-	-	-	-
T	102.650	10.999	100.150	9.051	99.450	9.023	0.356	0.837	-	-	-	-
PER	100.000	8.944	110.350	16.259	103.450	10.773	6.488	0.039^*^	0.034^*^	0.982	0.355	a < b
An	111.300	15.994	112.850	11.820	108.850	11.731	0.293	0.864	-	-	-	-

Significance: ^*^p < 0.05; ^**^p < 0.01. Groups: a, REDs; b, NSSIs; c, SAs. See Supplementary Table S1 for the legends of all R-PAS variables.

**Table 6:** ANCOVA and effect size concerning R-PAS indexes in the three groups.

	**F**	***p***	**ω^2^**	**REDs vs. NSSIs**	**REDs vs. SAs**	**NSSIs vs. SAs**	**Contrast**
				***p***	***p***	***p***	
**Page 1**							
**Administration. behaviors and observation**							
Pr	0.752	0.476	0.000	-	-	-	-
Pu	4.472	0.016^*^	0.102	0.025^*^	1.000	0.054	a > b
CT	0.155	0.857	0.000	-	-	-	-
**Engagement and cognitive processing**							
Complexity	1.323	0.275	0.011	-	-	-	-
R	1.650	0.201	0.021	-	-	-	-
F%	0.609	0.548	0.000	-	-	-	-
Blend	2.793	0.070	0.057	-	-	-	-
Sy	1.838	0.169	0.028	-	-	-	-
MC	0.419	0.660	0.000	-	-	-	-
MC-PPD	1.113	0.336	0.004	-	-	-	-
M	0.709	0.497	0.000	-	-	-	-
M/MC	0.546	0.583	0.000	-	-	-	-
(CF+C)/SumC	0.622	0.546	0.000	-	-	-	-
**Perception and thinking problems**							
EII-3	0.155	0.857	0.000	-	-	-	-
TP-Comp	0.076	0.926	0.000	-	-	-	-
WSumCog	1.445	0.245	0.015	-	-	-	-
SevCog	0.719	0.492	0.000	-	-	-	-
FQ-%	0.131	0.878	0.000	-	-	-	-
WD-%	0.008	0.992	0.000	-	-	-	-
FQo%	2.692	0.077	0.052	-	-	-	-
P	0.349	0.707	0.000	-	-	-	-
**Stress and distress**							
YTVC'	0.242	0.786	0.000	-	-	-	-
m	1.038	0.361	0.001	-	-	-	-
Y	0.395	0.676	0.000	-	-	-	-
MOR	2.777	0.071	0.056	-	-	-	-
SC-Comp	0.517	0.601	0.000	-	-	-	-
**Self and other representation**							
ODL%	2.090	0.133	0.035	-	-	-	-
SR	1.247	0.295	0.008	-	-	-	-
MAP/MAHP	NA	NA	NA	-	-	-	-
PHR/GPHR	0.293	0.748	0.000	-	-	-	-
M-	0.715	0.494	0.000	-	-	-	-
AGC	0.854	0.431	0.000	-	-	-	-
H	2.804	0.069	0.057	-	-	-	-
COP	2.316	0.108	0.043	-	-	-	-
MAH	1.336	0.271	0.011	-	-	-	-
**Page 2**							
**Engagement and cognitive processing**							
W%	0.768	0.469	0.000	-	-	-	-
Dd%	1.721	0.188	0.024	-	-	-	-
SI	0.771	0.467	0.000	-	-	-	-
IntCont	0.905	0.410	0.000	-	-	-	-
Vg %	2.897	0.064	0.060	-	-	-	-
V	1.076	0.348	0.003	-	-	-	-
FD	1.861	0.165	0.029	-	-	-	-
R8910%	0.104	0.901	0.000	-	-	-	-
WSumC	1.201	0.309	0.007	-	-	-	-
C	1.024	0.366	0.000	-	-	-	-
Mp/(Ma+Mp)	0.402	0.673	0.000	-	-	-	-
**Perception and thinking problems**							
FQu%	2.541	0.008	0.050	-	-	-	-
**Stress and Distress**							
PPD	0.259	0.773	0.000	-	-	-	-
CBlend	2.243	0.116	0.041	-	-	-	-
C'	2.783	0.071	0.058	-	-	-	-
CritCont%	3.141	0.051	0.069	-	-	-	-
**Self and other representation**							
SumH	0.437	0.648	0.000	-	-	-	-
NPH/SumH	4.296	0.019^*^	0.113	0.017^*^	1.000	0.168	a > b
V-Comp	0.360	0.699	0.000	-	-	-	-
r	0.062	0.940	0.000	-	-	-	-
p/(a+p)	0.239	0.788	0.000	-	-	-	-
AGM	2.050	0.138	0.034	-	-	-	-
T	0.540	0.586	0.000	-	-	-	-
PER	4.125	0.021^*^	0.097	0.020^*^	1.000	0.173	a < b
An	0.376	0.688	0.000	-	-	-	-

Significance: ^*^p < 0.05; ω^2^: very small (< 0.01), small (between ≤ 0.01 and < 0.06), medium (between ≤ 0.06 and < 0.14), large effect (≥0.14). Groups: a, REDs; b, NSSIs; c, SAs. See Supplementary Table S1 for the legends of all R-PAS variables.

## 4 Discussion

This study aimed to describe the typical psychological and behavioral characteristics of three adolescent patient groups who express psychological distress through their bodies.

Data suggests subthreshold OCD symptoms, apparently preserved functioning, and fanciful representations of interpersonal relationships as distinctive features of the RED group. The high comorbidity between REDs and obsessive-compulsive tendencies has been proven in previous studies (Mandelli et al., 2020) and, along with good functioning, suggests perfectionism traits and the need for control. This tendency is also reflected in the focus on minor details shown during the assessment. Moreover, most of those adolescents expressed depression symptoms and subthreshold separation anxiety. These findings are consistent with prior research highlighting the emotional burden associated with restrictive eating behaviors (Touchette et al., 2011; Zanna et al., 2021) and difficulties aligning their perceptions with common interpretations, reflecting an individualistic worldview (Rothschild et al., 2008; Guinzbourg de Braude et al., 2021).

The NSSI group reported chronic distress, reflected in subthreshold depressive symptoms. Moreover, they frequently exhibited subthreshold panic and social anxiety symptoms, consistent with existing literature (Bentley et al., 2015). Interestingly, none of the participants in the NSSI group showed suprathreshold symptoms on the K-SADS-PL subscales, which aligns with previous findings indicating that adolescents engaging in NSSI typically experience chronic distress without the immediate lethality often associated with SAs (Auerbach et al., 2021). Data also corroborated previous findings linking self-mutilating behavior with borderline personality characteristics (Stead et al., 2019; American Psychiatric Association, 2022), highlighting difficulties in emotion regulation, defensiveness and emotional distancing in relationships, and a lower sense of family cohesion, which are commonly observed in adolescents with borderline traits (Marrero et al., 2023).

Findings then showed that the SA group exhibited the highest frequency of K-SADS suprathreshold scores. Notably, most of these participants scored in the suprathreshold range for depression and presented the highest prevalence of emotional dysregulation, with profound difficulties in managing both internal states and external demands. Other distinctive symptoms were psychotic, social anxiety, and GAD. This is in line with existing research linking depression, anxiety, and substance abuse to SA (Alvarez-Subiela et al., 2022). Adolescents experiencing severe depressive episodes or life crises, often compounded by psychotic symptoms, typically show high levels of psychopathology, impulsivity, and significant functional impairment (Kelleher et al., 2013; Auerbach et al., 2021).

The second aim was to identify shared and distinct features across these three groups of patients using a multimethod assessment approach. The findings revealed similarities and distinctive differences across the groups, highlighting the complexity of these manifestations.

All groups showed comparable PTSD scores, consistent with previous literature (Bentley et al., 2015; Panagioti et al., 2015; Rijkers et al., 2019). Adverse childhood experiences were associated with NSSIs, SAs (Laporte et al., 2023), and eating disorders (Kovács-Tóth et al., 2022; Pauls et al., 2022), suggesting a shared underlying vulnerability in how early trauma shapes psychopathology. This stresses the importance of considering childhood trauma as a critical factor in the onset of such disorders. In line with existing literature, all groups exhibited similar patterns of avoidant and obsessive-compulsive personality traits (Laczkovics et al., 2023), reflecting an association with dysfunctional attachment styles (Braga and Gonçalves, 2014; Amianto et al., 2022).

Moreover, the assessment delineated disrupted reality-testing and disorganized thinking in all groups, aligning with previous studies regarding individuals with AN (Rothschild et al., 2008), NSSI, and SA (Auerbach et al., 2021). Similarly, data suggested pervasive distortions in interpreting familiar and simple stimuli, reflecting a significant cognitive and perceptual processing impairment. This pattern suggests difficulties aligning perceptions with commonly accepted interpretations and difficulties in emotional regulation and interpersonal relationships. Consistently, tests showed the presence of physical, medical, or body-related concerns from all the groups, as previous literature found (Muehlenkamp and Brausch, 2012; Brunner et al., 2014; Cipriano et al., 2017; De Luca et al., 2023; Mirabella et al., 2023).

Regarding distinctive differences, findings showed more borderline personality traits in NSSIs, especially compared to the RED group, confirming literature that links self-injury with borderline personality traits, emotion regulation challenges, and interpersonal difficulties (Stead et al., 2019). However, the RED group showed the highest productivity levels and perfectionism, maybe to protect themselves against anxiety or insecurity. Even if the NSSI group displayed higher impressionistic, evasive, and defensive responses compared to the SA group, and a higher attraction to ambiguity and traumatic experiences than REDs, confirming emotional regulation difficulties (Braga and Gonçalves, 2014; Auerbach et al., 2021). This reflects the tendency to loneliness, progressive isolation (Li et al., 2024), and the use of self-harm as a maladaptive strategy for regulating intense emotions and social challenges during stressful events (Hou et al., 2023). These interpretations are consistent with studies suggesting that early adverse experiences, such as emotional neglect, predispose individuals with NSSI to heightened aversive emotions, poor distress tolerance, and impaired social skills. Finally, the SA group had the worst overall functioning, with shared impairments with the other group, confirming that SA is transdiagnostic.

Regarding the study limits, we should mention that the participants were self-selected. They consented to participate in the study from a larger cohort of patients. Furthermore, given that patients came mainly from Northern Italy, the possibility of generalizing results to the general population is reduced. Another limitation is the absence of data regarding participants' sexual orientation and gender identity, as the study protocol did not include this information for research purposes. Given the established associations between sexual minority status and increased risk of NSSI, eating disorders, and body dissatisfaction (Batejan et al., 2015; Liu et al., 2019; Rezeppa et al., 2021; Muzi et al., 2023), future research should further explore the role of these factors to enhance the generalizability and clinical applicability of findings in diverse adolescent populations. We then excluded patients with more than one type of self-directed physical harm. Accordingly, we lowered the presence of potential biases, resulting in a low sample size. This may have limited the analyses' statistical power and the ability to detect finer effects. Moreover, participants were assigned to one of the three groups based on their primary clinical presentation but due to the high comorbidity rates in adolescent psychopathology, some participants may have exhibited symptoms of other psychiatric conditions, which were recorded but not used for group classification. Future studies may benefit from considering dimensional approaches or transdiagnostic models to capture the complexity of co-occurring symptoms better. Furthermore, the sample was inhomogeneous due to the prevalence of females. This reflects the higher presence of psychiatric disorders in female adolescents, as well as the fact that females are more likely to ask for help than males (Dil et al., 2024). The small number of male participants limits the data' generalizability to the larger adolescent population. This gender disparity emphasizes the need for future research to have more equitable gender representation. Moreover, the study lacks a healthy control group, and future studies should include one to highlight differences between clinical and non-clinical conditions. Finally, the cross-sectional design does not allow for causal inferences.

To conclude, this study explored psychological and behavioral characteristics among adolescents manifesting psychological distress through their bodies, highlighting potential differences across groups. The RED group was characterized by pronounced depressive and OCD symptoms, coupled with perfectionism and distorted self-representations. The NSSI group, on the other hand, presented with subthreshold depressive symptoms, marked borderline personality traits, and significant emotion regulation deficits, which were mirrored in their defensive and impressionistic patterns on the R-PAS. The SA group emerged as the most clinically severe, exhibiting heightened depressive and dysregulation symptoms, impaired functioning, and notable challenges in thought organization. While these differences highlight the unique features of each group, commonalities also emerged, including pervasive distortions in interpreting stimuli and shared associations with adverse childhood experiences. By integrating multiple assessment tools, including the R-PAS, this study provided a nuanced understanding of the interplay between symptomatology, personality traits, and cognitive processes in these populations.

Understanding these distinct and overlapping features has crucial therapeutic and clinical practice implications. Given the centrality of emotional dysregulation across all three groups, therapeutic approaches such as psychodynamic psychotherapies or Cognitive-behavioral Therapy (CBT) (Beck, 2021) should prioritize emotion regulation strategies. Another promising therapeutic approach, initially developed specifically for those suffering from borderline personality disorders, is Dialectical Behavior Therapy (DBT) (Linehan, 2015), which has been found effective across different disorders involving difficulties controlling emotions, self-criticism, and interpersonal issues. Psychodynamic and cognitive-behavioral approaches should emphasize crisis intervention, reality testing, and strengthening self-cohesion. The findings also suggest that a body-focused approach may benefit all three groups. Techniques such as mindfulness, somatic therapies, and interventions addressing body image disturbances could help adolescents develop healthier relationships with their bodies and emotions (Van der Kolk, 2015). Hence, working on trauma could improve patients' outcomes and interpersonal relationships. Moreover, given the impact of social media on body image and self-harm behaviors (Memon et al., 2018; Hornberger et al., 2021), psychoeducational interventions should be integrated into prevention strategies, even in non-clinical settings. Furthermore, systemic psychotherapies and family-based treatment (FBT) included in multidisciplinary approaches could help patients and their families (Mensi et al., 2021), improving cognitive rigidity and perfectionism while promoting flexible thinking, interpersonal relationships, and emotional awareness.

These findings reinforce the importance of personalized, transdiagnostic approaches to adolescent mental healthcare, tailoring interventions to adolescents' specific distress manifestations. Recognizing and addressing distinct symptom patterns and underlying psychological mechanisms can inform the development of more effective, personalized therapeutic approaches. Such efforts are crucial for improving outcomes and addressing the multifaceted needs of adolescents experiencing psychological distress.

## Data Availability

The datasets presented in this study can be found in online repositories. The names of the repository/repositories and accession number(s) can be found below: Zenodo (10.5281/zenodo.14547866).
