# Supplementary material for: Body as expression of psychiatric distress: insights from restrictive eating disorders, non-suicidal self-injuries, and suicide attempts
Source: Front Psychol. 2025 Mar 14;16:1552907. doi: 10.3389/fpsyg.2025.1552907 (PMC11949972; doi:10.3389/fpsyg.2025.1552907)
Supplement: Supplementary file 2 [file Table_2.docx]

Table S1. Legend of all R-PAS variables

| Pr SS | Prompt Standard Score |
| --- | --- |
| Pu SS | Pull Standard Score |
| CT SS | Card Turning Standard Score |
| Complex SS | Complexity Standard Score |
| R SS | Responses Standard Score |
| F% SS | Simplicity Standard Score |
| Blend SS | Blend Standard Score |
| Sy SS | Synthesis Standard Score |
| MC SS | Human movement and the weighted sum of color determinants Standard Score |
| MC-PPD SS | MC- potentially problematic determinants Standard Score |
| M SS | Human Movement Standard Score |
| M/MC SS | Proportion of Human movement and Human movement and the weighted sum of color determinants Standard Score |
| (CF+C)/SumC SS | Proportion of with Color and Form and only Color responses Standard Score |
| EII-3 SS | Ego Impairment Index- 3 Standard Score |
| TP-Comp SS | Thought & Perception Composite Standard Score |
| W SumCog SS | Weight Sum of Cognitive Coding Standard Score |
| SevCog SS | Weight sum of Severe Cognitive Coding Standard Score |
| FQ-% SS | Form Quality minus Standard Score |
| WD-% SS | Whole or Detailed response with form quality minus Standard Score |
| Fqo% SS | Form Quality Ordinary Standard Score |
| P SS | Popular responses Standard Score |
| YTVC' SS | Sum of light-dark and achromatic determinants Standard Score |
| m SS | Inanimate movement Standard Score |
| Y SS | Light-dark responses Standard Score |
| MOR SS | Deteriorated content Standard Score |
| SC-Comp SS | Suicide Concern Composite Standard Score |
| ODL% SS | Oral Dependency Language Standard Score |
| SR SS | Space reversal Standard Score |
| MAP/MAHP SS | Proportion of Responses with Mutual of Autonomy Pathological Standard Score |
| PHR/GPHR SS | Proportion of Poor or Good Human Representation Standard Score |
| M- SS | Human movements with form quality minus Standard Score |
| AGC SS | Aggressive contents Standard Score |
| H SS | Human Content Standard Score |
| COP SS | Cooperative responses Standard Score |
| MAH SS | Mutual of Autonomy Healthy Standard Score |
| W% SS | Whole Standard Score |
| Dd % SS | Unusual Detail Standard Score |
| SI SS | Space Integration Standard Score |
| IntCont SS | Intellectualized Content Standard Score |
| Vg % SS | Vague Standard Score |
| V SS | View Standard Score |
| FD SS | Dimension form Standard Score |
| R8910% SS | Proportion of responses at tables VIII, IX, X Standard Score |
| WSumC SS | Weighted sum of color determinants Standard Score |
| C SS | Color Standard Score |
| Mp/(Ma+Mp) SS | Proportion of passive Human movement Standard Score |
| FQu% SS | Form Quality unusual Standard Score |
| PPD SS | Potentially problematic determinants Standard Score |
| Cblend SS | Sum of the blend of achromatic/light dark and color determinants Standard Score |
| C' SS | Achromatic color Standard Score |
| V SS | View Standard Score |
| CritCont% SS | Critical Contents Standard Score |
| SumH SS | Sum of Human Content Standard Score |
| NPH/SumH SS | Proportion of not pure H responses Standard Score |
| V-Comp SS | Vigilance Composite Standard Score |
| r SS | Reflections Standard Score |
| p/(a+p) SS | Proportion of passive Movement Standard Score |
| AGM SS | Aggressive Movement Standard Score |
| T SS | Texture Standard Score |
| PER SS | Personal responses Standard Score |
| An SS | Anatomy Standard Score |
